# Clinical and Histological Evaluation of Direct Pulp Capping on Human Pulp Tissue Using a Dentin Adhesive System

**DOI:** 10.1155/2016/2591273

**Published:** 2016-10-10

**Authors:** Alicja Nowicka, Ryta Łagocka, Mariusz Lipski, Mirosław Parafiniuk, Katarzyna Grocholewicz, Ewa Sobolewska, Agnieszka Witek, Jadwiga Buczkowska-Radlińska

**Affiliations:** ^1^Department of Conservative Dentistry and Endodontics, Pomeranian Medical University, Szczecin, Poland; ^2^Department of Preclinical Conservative Dentistry and Preclinical Endodontics, Pomeranian Medical University, Szczecin, Poland; ^3^Department of Forensic Medicine, Pomeranian Medical University, Szczecin, Poland; ^4^Department of General Dentistry, Pomeranian Medical University, Szczecin, Poland; ^5^Department of Gerodontology, Pomeranian Medical University, Szczecin, Poland

## Abstract

*Objective*. This study presents a clinical and histological evaluation of human pulp tissue responses after direct capping using a new dentin adhesive system.* Methods*. Twenty-eight caries-free third molar teeth scheduled for extraction were evaluated. The pulps of 22 teeth were mechanically exposed and randomly assigned to 1 of 2 groups: Single Bond Universal or calcium hydroxide. Another group of 6 teeth acted as the intact control group. The periapical response was assayed, and a clinical examination was performed. The teeth were extracted after 6 weeks, and a histological analysis was performed. The pulp status was assessed, and the thickness of the dentin bridge was measured and categorized using a histological scoring system.* Results*. The clinical phase was asymptomatic for Single Bond Universal patients. Patients in the calcium hydroxide group reported mild symptoms of pain, although the histological examination revealed that dentin bridges with or without limited pulpitis had begun forming in each tooth. The universal adhesive system exhibited nonsignificantly increased histological signs of pulpitis (*P* > 0.05) and a significantly weaker thin mineralized tissue layer (*P* < 0.001) compared with the calcium hydroxide group.* Conclusion*. The results suggest that Single Bond Universal is inappropriate for human pulp capping; however, further long-term studies are needed to determine the biocompatibility of this agent.

## 1. Introduction

Direct pulp capping is one of the oldest known treatments for exposed pulp, and more efficient materials and approaches are continuously sought [1–4]. The material used for pulp capping after exposure may affect the vitality and healing of the pulp [5]. The introduction of calcium hydroxide (Ca[OH]_2_) in dentistry has played an important role in the development of a biological treatment for exposed pulp, because of its potent antibacterial properties and its ability to stimulate reparative dentin formation, markedly increasing the success rates of clinical procedures [1, 6–9]. However, Ca(OH)_2_ does not adhere to dentin and dissolves over time; thus, it may fail to provide an effective long-term barrier against bacterial penetration [10].

New pulp capping techniques may stimulate pulp healing without toxic chemical effects, thus providing better results than Ca(OH)_2_ [2–4, 11]. Dentin adhesive systems have been investigated in humans [1, 2, 6, 9, 12, 13] and animals [7, 8, 14–16] as potential direct pulp capping materials because of their superior ability to adhere to demineralized dentin tissues. Hybridization of dentin bonding and diffusion of adhesives into the dentin tubules may protect the dentin against bacterial leakage and thereby reduce secondary pulpal inflammation [12]. Studies that compared the pulp response to dentin adhesive systems and Ca(OH)_2_ showed that adhesive systems did not significantly differ from Ca(OH)_2_, in most cases did not result in inflammation, and also induced dentin bridge formation [2, 13, 16].

However, other studies demonstrated leakage through nanometric spaces within the hybrid layer and have revealed cytotoxicity of components of the adhesive systems, such as bisphenol A-glycidyl methacrylate (Bis-GMA), urethane dimethacrylate, triethylene glycol dimethacrylate, camphorquinone, and 2-hydroxyethyl methacrylate (HEMA) [17, 18]. These monomers were shown to be able to diffuse through the dentin tubules and reach the pulpal space; long periods of exposure of cells to these monomers resulted in significantly increased cytotoxicity [17, 19, 20]. Self-etching bonding agents do not require prior total etching, and self-etching primers are less acidic than phosphoric acid; these can therefore provide a more favorable response than the etch-and-rinse systems [18, 19]. Kitasako et al. [21] demonstrated that the self-etching adhesive system, Clearfil Liner Bond 2V, allowed pulp healing and tertiary dentin deposition. The bonding agent was supposed to seal the exposure site and was effective as the barrier in the dentin bridges after bacterial challenge [2, 11, 21]. A recent study [22] showed that a one-step system had the best bond strength performance and was the least toxic to pulp cells.

Single Bond Universal (3M ESPE, Seefeld, Germany) is an “ultramild” self-etching adhesive (pH 2.7), which is a representative of the next generation of bonding systems available to dentists, the so-called “universal adhesives” (Table 1). It can be used for bonding not only to enamel and dentin, but also to ceramics, metal, and composites [20, 22, 23]. Single bond universal was the first universal adhesive, which reached high mean microtensile dentin bond strengths [22, 23]. Application of this bonding system to the dentin surface results in the formation of a hybrid layer, with superior chemical bonding of the monomer 10-methacryloyloxydecyl dihydrogen phosphate (10-MDP) to hydroxyapatite [24]. An additional chemical mechanism is related to the interaction of a polyalkenoic acid copolymer with the calcium in hydroxyapatite. This self-assembled nanolayering of two 10-MDP molecules, joined by a stable MDP-Ca salt formation, makes the adhesive interface more resistant to biodegradation [22, 24].

Despite Single Bond Universal's frequent clinical use, the effects of its system on human pulp have not yet been reported. In vivo studies on humans are required to understand the pulp response when adhesive systems are used as direct pulp capping. The present study performed clinical, radiographic, and histological evaluations of the human pulp responses after direct capping with Single Bond Universal. The null hypothesis was a lack of difference in the response of the pulp tissue between the two direct pulp capping techniques (universal adhesive system versus calcium hydroxide) for human teeth.

## 2. Materials and Methods

The study was conducted in accordance with the tenets of the Declaration of Helsinki. Twenty-eight caries-free, intact, maxillary, and mandibular third molars from 17 humans, aged 19–30 years, which were scheduled for extraction for orthodontic or surgical purposes, were included in the study. The patients received a thorough explanation of the experimental rationale, clinical procedures, and possible complications. All experimental protocols were approved by the Local Ethics Committee of Pomeranian Medical University, Szczecin, Poland (approval number KB–0012/39/11).

### 2.1. Operative Procedure

Standardized therapeutic procedures were used. Each tooth was radiologically examined to exclude the presence of caries or periapical pathology. Thermal testing (Kältespray, M&W Dental, GmbH, Büdingen, Germany) and electric sensitivity testing (Vitality Scanner pulp vitality tester, SybronEndo, Orange, CA) were performed to determine tooth sensitivity. Before cavity preparation, the teeth were mechanically cleaned and disinfected with 0.2% chlorhexidine solution. Following the induction of local anesthesia and application of a rubber dam, occlusal Class I cavities were prepared using a sterile round diamond bur at a high speed, under air-distilled water cooling. An area measuring approximately 1.2 mm in diameter was exposed using round carbide burs (ISO size 012) under air-distilled water cooling in the center of the pulp floor. New burs were employed for each procedure. Bleeding was controlled with saline irrigation and a sterile cotton pellet. All cavities and pulp exposures were performed by the same clinician.

The teeth were randomly divided into 3 groups and treated as follows.

1. Control intact group (IT): 6 teeth with no exposure and pulp capping.
2. Control group (CH): 11 teeth capped with Ca(OH)_2_ paste Calcipast (Cerkamed, Stalowa Wola, Poland), followed by Life (Kerr Hawe, Salerno, Italy), and Single Bond Universal with Filtec Ultimate (3M ESPE, Seefeld, Germany) according to the manufacturer's recommendations.
3. Experimental group (SBU): 11 teeth capped with Single Bond Universal with Filtec Ultimate, according to the manufacturer's recommendations; Single Bond Universal was applied to the exposed pulp and cavity walls for 20 s and light-cured for 10 s.

The same operator restored all of the cavities.

### 2.2. Clinical Examination

The pulp status was evaluated based on patient-reported symptoms using a verbal pain intensity scale, clinical examinations, and thermal and electrical tests. Immediately after filling the cavity we asked patients to register their discomfort after surgery and coming to control visit. The clinical assessment was performed after 1 week and after 6 weeks of observation, directly before tooth extraction. During the clinical interview, the patient was asked about the possible pain, their type and duration. We use a verbal pain intensity scale, which is a list of verbal descriptors defining pain, such as the following: absent, weak, moderate, and strong. The patients chose the word that best described their pain. To analyze the duration of pain, we used the following classification: 0, no pain; 1, symptoms lasting for one day; 2, symptoms lasting for 2–7 days; 3, symptoms lasting longer than 7 days. Reaction to thermal stimuli was classified into three categories: 1, normal positive reaction (pain lasting up to 10 s); 2, an extended positive reaction (pain > 10 s); 3, no reaction. An electrical test was carried out using a Vitality Scanner. This test was repeated three times.

Radiographs were taken six weeks after the application of the pulp capping material in order to evaluate the status of the periapical tissues, presence of deposits in the pulp chamber, and the presence of internal or external resorption. Subsequently, a designated oral surgeon extracted the teeth with minimal trauma.

### 2.3. Histological Assessment

All extracted teeth were evaluated for microscopic pulpal response. The evaluation was carried out as a blind test by two persons. Compliance was excellent. Coded samples were used throughout the study to avoid possible bias. The extracted teeth were fixed in 10% buffered formalin solution for 2 weeks, then demineralized in nitric acid, and embedded in paraffin. Two- to 3-micron thick serial sections, cut in the lingual-buccal plane, were stained with hematoxylin and eosin (H&E). Bacteria were detected using the Brown and Brenn technique.

Histological analyses were performed on the H&E-stained specimens using an optical microscope (Carl Zeiss Imager D1 Axio, Goettingen, Germany) connected to a high-resolution video camera (AxioCam MRc5,* Carl Zeiss* Microimaging, Thornwood, NY). An experienced examiner investigated the sections under normal and ultraviolet (UV) light using 38 HE eGFP (which clearly illustrated the physiological secondary dentin and the original dentin) and 43 HE Cy3 filters (clearly illustrating the kernel odontoblasts and blood cell counts). The amount of hard tissue formed at the interface of the capping material was analyzed using the criteria presented in Table 2. The histological sections were given scores ranging from 1 to 4, with 1 representing the most desired result and 4 representing the least desired result. All sections of the examined teeth were assessed. Calculations were made from sections of the thickest dentin bridge. The thickness of the dentinal bridge was measured at the thickest, thinnest, and midmost point areas of the bridge. The average of the 3 values was then calculated.

In order to assess the number of inflammatory cells for each tooth, all analysis sections were selected from areas with the largest number of inflammatory cells. In these localized areas, using a magnification of 400x the number of inflammatory cells within the field of view was calculated, and the average value was determined for each tooth.

### 2.4. Statistical Analysis

The normal distribution of all continuous variables was verified using the Kolmogorov-Smirnov test. The data were subjected to the Mann-Whitney *U* test and Spearman's rank test. Differences were considered significant at *P* < 0.05.

## 3. Results

### 3.1. Clinical Examination

None of the patients in the SBU group reported any particular symptoms during the experimental time period, although 2 patients in the CH group complained of spontaneous minor pain and 1 patient complained of moderate pain, mostly on the day of surgery.

Teeth from all of the groups responded positively to electric pulp testing and were cold sensitive prior to extraction. Radiography revealed no periapical pathologies before extraction.

### 3.2. Histological Assessment

**Figure 1:**
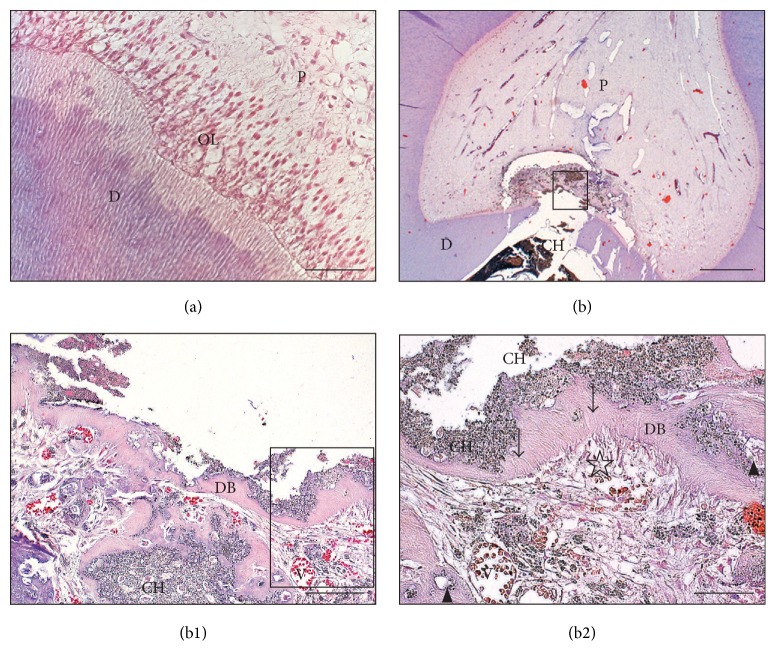
Morphology of the pulp-dentin complex in control groups. (a) Control intact group. Normal pulp tissue with visible odontoblast cell layer (hematoxylin and eosin [H&E]). Scale bar = 50 *μ*m. (b) View of the pulp-dentin complex after application of calcium hydroxide onto the exposed pulp. Complete dentin bridge and no inflammatory process in the remaining pulp (H&E). Scale bar = 1 mm. (b1) Fragment of the view seen in panel (b). Dentin bridge with tunnel defect (*arrowhead*) and particles of calcium hydroxide in the pulp and bridge (H&E). Scale bar = 200 *μ*m. (b2) Fragment of the view seen in panel (b1). Dentin bridge with dentinal tubules (arrow) and new odontoblast cell layer (star) (H&E). Scale bar = 100 *μ*m. D: dentin; DB: dentin bridge; OL: odontoblast layer; P: pulp; V: blood vessels.* Arrow:* dentinal tubules;* arrowhead*: tunnel defect;* star*: new odontoblast cell layer.

The control intact teeth exhibited an odontoblast layer, a zone of Weil, a cell-rich zone, and central pulp with normal characteristics, with no inflammatory cells present (Figure 1(a)).

**Figure 2:**
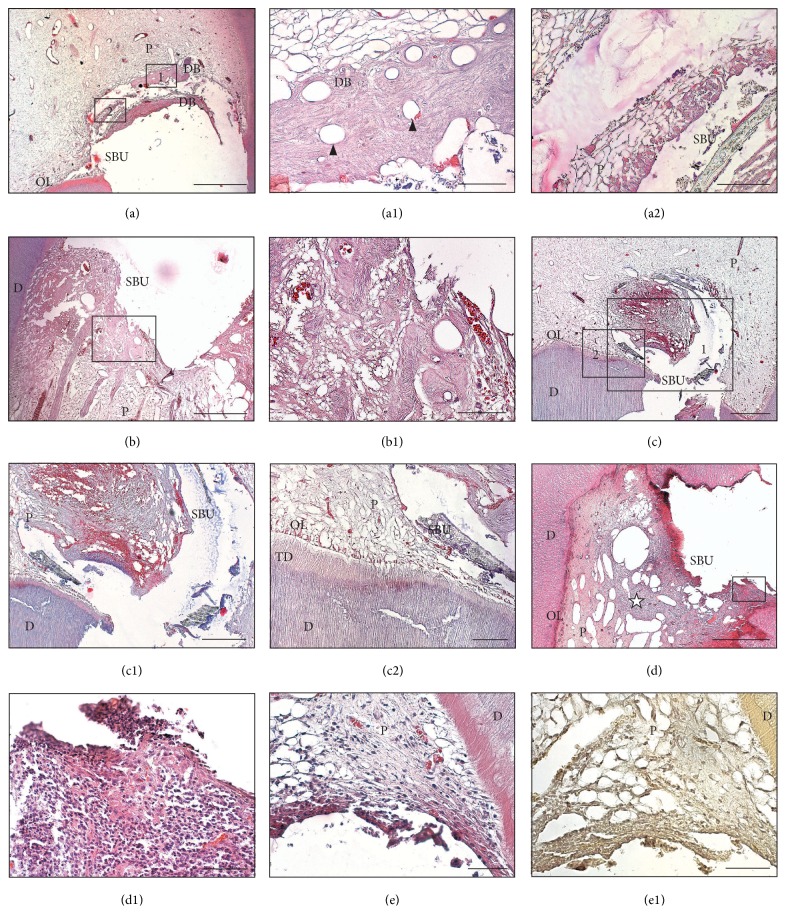
Human pulp capped with Single Bond Universal. (a) Incomplete dentin bridge formation and no inflammatory process visible in the remaining pulp (hematoxylin and eosin [H&E]). Scale bar = 400 *μ*m. (a1) Fragment of the view seen in frame 1 of panel (a). The dentin bridge with tunnel defect (*arrowhead*) is observed. Scale bar = 50 *μ*m. (a2) Fragment of the view seen in frame 2 of panel (a). A layer of the bonding system is observed under the tissue of the bridge. Scale bar = 50 *μ*m. (b) No dentin bridge. Pulp fibrosis at the site of contact with the Single Bond Universal (H&E). Scale bar = 300 *μ*m. (b1) Fragment of the view seen in panel (b). Tissue resembling fibrodentin. Scale bar = 50 *μ*m. (c) No dentin bridge (H&E). Scale bar = 600 *μ*m. (c1) Fragment of the view seen in frame 1 of panel (c). Hemorrhagic infiltration and presence of the material in the pulp. Scale bar = 350 *μ*m. (c2) Fragment of the view seen in frame 2 of panel (c). Thinning of the odontoblast layer and tertiary dentin near the site of exposure. Scale bar = 150 *μ*m. (d) No dentin bridge. Pulpitis with rich infiltrate of leukocytes (star) and marked dilation of blood vessels (H&E). Scale bar = 500 *μ*m. (d1) Fragment of the view seen in panel (d) with amorphous areas of necrosis and rich infiltrate of leukocytes. Scale bar = 100 *μ*m. (e) No dentin bridge. Chronic pulpitis (H&E). Scale bar = 100 *μ*m. (e1) View of the preparation stained according to Brown and Brenn (B&B). No positive staining for bacteria. Scale bar = 100 *μ*m. D: dentin; DB: dentin bridge; OL: odontoblast layer; P: pulp; TD: tertiary dentin;* arrowhead*: tunnel defect;* star*: infiltrate of leukocytes.

**Figure 3:**
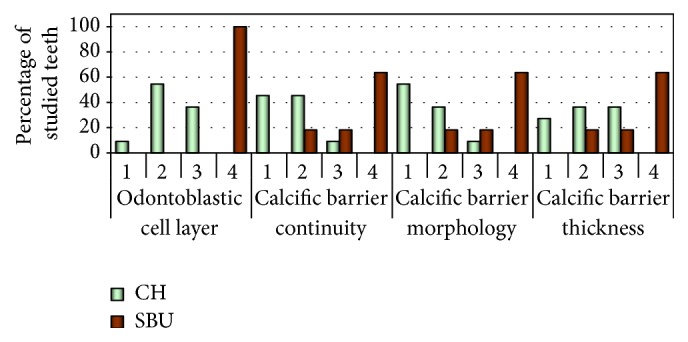
Odontoblastic cell layer occurrence and calcific barrier continuity, morphology, and thickness after direct pulp capping in the study groups (*P* < 0.001). CH: calcium hydroxide; SBU: Single Bond Universal.

Calcium hydroxide actively initiated the formation of reparative dentin with varying levels of organization in each tooth (Figures 1(b)–1(b2)); however, Single Bond Universal was significantly less active and induced the formation of total 4 bridges (2 small and 2 very small) (Figures 2(a)-2(a1) and 3). The reparative tissue seen in the CH group was usually in the form of dentin laid down next to irregular mineralized tissue. In most specimens in the CH group, odontoblasts and odontoblast-like cells were visible in the tooth (Figures 1(b1)-1(b2) and 3). In the SBU group, reparative tissue showed fibrodentin and osteodentin occurring in a small layer, in the absence of a new odontoblast layer (Figures 2(a)-2(a1)). At high magnification, the reparative hard tissues in both groups exhibited porosities and tunnel defects (Figures 1(b1)-1(b2) and 2(a1)). The thickness of the dentin bridges in the SBU group was significantly lower (*P* < 0.001) than that in the CH group. The average maximum thickness of the dentin bridges in the SBU group was 54.4 *μ*m, compared to the overall average of 30.3 *μ*m; in the CH group, these were 259.4 *μ*m and 182.3 *μ*m, respectively (Figure 3). In most sections of the CH and the SBU groups, a small or moderate amount of material particles of Ca(OH)_2_ and Single Bond Universal was observed in the pulp, respectively (Figures 1(b)–1(b2) and 2(a), 2(a2), 2(c), 2(c1), and 2(c2)).

**Figure 4:**
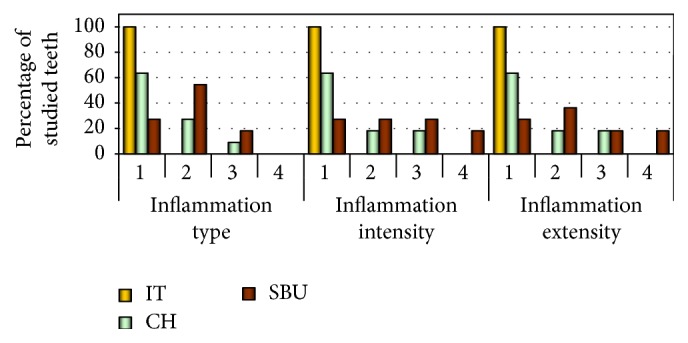
Pulp inflammatory reaction after direct pulp capping in the study groups (type, intensity, and extent). IT^*∗*^: control intact group; CH: calcium hydroxide group; SBU^*∗*^: Single Bond Universal group; (*∗*) significant difference between groups (*P* > 0.005).

There were no statistically significant differences in terms of the type, intensity, and coverage of the inflammatory response of the pulp between the SBU and the CH groups. There were also no statistically significant differences between the CH and the IT groups, but these differences were significant between the SBU and the IT groups (Figure 4). No significant differences between the CH and the IT groups indicate good pulpal compatibility of the material. In the CH group, 7 of the teeth showed no evidence of inflammation, and 4 of the teeth revealed chronic inflammation. In the SBU group, chronic inflammation of the pulp and total increased tissue reaction were common, with widening and expansion of the capillaries, as shown in Figures 2 and 4. The inflammatory response in the SBU group was localized and limited to the area of the pulp in contact with the composite in 4 teeth, reached the middle of the coronal pulp in 2 teeth, and covered the entire coronal pulp in 2 additional teeth. In some preparations, less vascular reaction was observed, while the reaction from the connective tissue was greater (Figures 2(b)-2(b1)). Atrophy and disorganization of the odontoblasts layer were observed in the vicinity of the exposed space (Figure 2(c2)). In 2 cases, acute and chronic inflammation with superficial necrosis and extensive infiltration of leukocytes were found (Figures 2(d)–2(e)).

Bacteria were not observed on the walls, tubular dentin, or pulp tissue in any of the teeth (Figure 2(e1)).

## 4. Discussion

Clinical and histological evaluation is the gold standard for the assessment of pulp reaction to the test material; therefore, in vivo studies are required to understand pulp response when dental materials are used for direct pulp capping [1, 12, 25]. The present investigation performed on humans was aimed at providing direct information about the biocompatibility of the universal adhesive system. Histologically, Single Bond Universal demonstrated significantly weaker initiation of the formation of a thin layer of mineralized tissue and nonsignificantly more intense inflammation of the pulp than did calcium hydroxide, despite asymptomatic clinical phase. Therefore, the null hypothesis of no differences in the pulp tissue response with the use of these 2 direct pulp capping techniques for human teeth cannot be accepted.

In this study, we used strict criteria for the classification of patients for surgery. The patients were young and healthy. The size of the treatment groups was limited to 11 teeth due to the difficulty in obtaining research material and this number was comparable with other studies on humans [6, 26]. The numbers of teeth in each group were reasonably and evenly distributed in terms of age and site of exposure. Specific operating procedures used during pulp capping such as controlling bleeding are critical for successful direct capping [26–28]. In the present study, pulp bleeding was controlled only through irrigation with saline solution and the placement of sterile cotton pellets onto the pulp exposure sites. All the cases achieved hemostasis. Other authors also describe the use of saline solution for hemostasis as being nontoxic and effective [6, 9, 12, 26–28].

One-step self-etching systems are considered a good alternative for Ca(OH)_2_, mainly due to its technical simplicity and good biological responses [13, 18]. Single Bond Universal elicited pulp responses similar to those observed with the use of dentin adhesive systems in humans [12, 13, 16]. Silva et al. [12] applied Scotchbond Universal, using the total etch technique during the clinical procedure of direct pulp capping, and revealed subclinical adhesive failure despite the satisfactory visual results. At the margins of the exposed pulp, residual monomer, blood, adhesive eruptions, globules, and gaps between the adhesive layers were observed. Excess humidity from the exposed pulp may have interfered with the polymerization of the first layer of adhesive, although the upper layers were clinically satisfactory and all care was taken to minimize monomer polymerization errors [12]. The residual monomer of bonding material can be cytotoxic, inhibit proliferation of immunocompetent cells, and induce immunosuppression that favors the development of pulp pathologies, despite the absence of bacteria [19].

In recent study by Kim et al. [29], the minimally toxic concentrations of 10-MDP caused the release of inflammatory cytokines including nitric oxide, prostaglandin E_2_, inducible nitric oxide synthase, COX-2 protein, TNF-*α*, IL-1*β*, IL-6, and IL-8 and suppressed odontoblastic differentiation of dental pulp cells by activating nuclear factor 2-mediated heme oxygenase-1 induction. The 10-MDP was found to be greater in inhibiting cellular proliferation than TEGDMA [29]. Monomers such as TEGDMA, HEMA, and 10-MDP appeared to contribute to the inflammation and inhibition of odontoblastic differentiation also by inducing oxidative stress via the production of reactive oxygen species and reduction of intracellular glutathione levels [17, 19, 29, 30]. Moreover, the combination of Bis-GMA, dimethacrylate, and/or polyalcene acids in the bonding system may play a role in the apparent cytotoxicity of this product and may suppress the healing capacity of the pulp tissue [7, 31]. In a study by Lee et al. [32], the absence of complete bridging in a group treated with TheraCal was attributed to the lower biocompatibility of the material, which caused a higher degree of inflammation due to the acrylic monomer Bis-GMA present in the material.

It is known that, with a higher degree of conversion, the rate of release of unpolymerized monomers is lower [20]. Despite these guidelines, complete curing of the direct capping material is difficult to achieve in vivo, and the uncured monomer has cytotoxic effects on pulpal cells [17–20, 33, 34]. Not only do the degree of conversion and monomer release determine the biocompatibility of adhesives, but the cytotoxicity of the photoinitiator should also be taken into account, as these reactive compounds are not bound to the matrix and may leach from the adhesives [20]. Camphorquinone, which is included in Single Bond Universal at millimolar concentrations, induces oxidative DNA damage and generation of reactive oxygen species [35].

Similarly, other studies have shown that, despite the good sealing and satisfactory biocompatibility of dentin adhesive systems, their capacity of inducing dentin repair was significantly weaker than that of Ca(OH)_2_ [2, 13, 16]. Other studies [1, 8] have reported total absence of continuous hard tissue bridge formation after using a bonding system for pulp capping. The reaction rate of calcification can be an important factor in vital pulp therapy agents [32]. The authors consider that direct pulp capping should generate a complete dentin bridge at the pulpal exposure area, to prevent invading bacteria and keep the pulp stable [7, 9, 10, 12, 14, 16]. Incomplete amorphous dentin bridge formation in the SBU group, with varying degrees of mineralization, occurred in only 4 specimens. Although Ca(OH)_2_ induced a less intense inflammatory response and more consistent formation of reparative dentin, both materials induced the formation of a porous reparative dentin bridge, with tunnel defects; therefore, the pulp is not completely sealed from the environment, because the bridges remain permeable. Therefore, when bonding of resins to the underlying tissue deteriorates over time, the risk of pulp infection is increased, and thus the use of these materials on exposed pulp should be carefully considered [10, 36]. Resin hydrolysis and enzymatic degradation of collagen may negatively affect the long-term bonding stability, regardless of the bonding strategy employed [33, 37].

In recent studies [29, 30], the resin monomer inhibited expression of proteins characteristic of the odontoblastic phenotype of dental pulp cells. Their findings suggested that dimethacrylate induced downregulation of mineralization-related genes (such as those encoding collagen *α*(1)I, ALP, bone sialoprotein, osteocalcin, RUNX2, and dentin sialophosphoprotein), resulting in reduced mineralization activity and calcium deposition [29, 30]. This may explain the failure of reparative dentin formation after direct pulp capping with bonding agents. Understanding the mechanisms underlying adaptive cell responses is necessary for the development of smart dental restorative materials and effective strategies for use in direct pulp therapy [17, 29]. It has been suggested that dentin bonding agents be combined with other chemicals (e.g., N-acetyl cysteine, simvastatin, CaCl_2_, dentin matrix protein 1, and dentin sialophosphoprotein) to promote odontogenic differentiation of pulp cells [15, 38–40].

When choosing an adhesive system for clinical use, its biocompatibility should be taken into consideration [18]. The present study was conducted under controlled experimental conditions, using human teeth, to avoid the impact of confounding factors. The teeth had healthy pulp, so that differences encountered in the pulp reaction could be attributed exclusively to the capping material used. The intensity of the pulp-dentin complex reactions demonstrated in healthy teeth may be lower than those observed in carious teeth [41].

Specimens in our study reflect only a 6-week snapshot. The 6-week follow-up period was sufficient to establish tentative prognoses [26]. The similar observation period was used by other authors in humans [9, 12, 26]. We cannot surmise whether equal histological healing could occur over an extended period of time; therefore, long-term studies are needed to verify the biocompatibility of the Single Bond Universal system [12, 13, 16].

Based on previous studies [3, 4, 25, 32], other new methods and materials, such as ProRoot MTA, Biodentine, Endocem, and RetroMTA, are more effective than Ca(OH)_2_ and dentin adhesive system for regeneration of the pulp-dentin complex with limited inflammation. A recent study [42] evaluated the clinical outcomes of treatment of exposed pulp with poly(*ε*-caprolactone) fiber mesh (PCL-FM) as a barrier for MTA. Exposed pulp, separated by the PCL-FM, created a favorable surface for pulp cell attachment, proliferation, and further differentiation into odontoblast-like cells, which in turn formed a thicker dentin bridge [4]. Tricalcium silicate cement was shown to stimulate osteogenic/odontogenic capacity after direct pulp capping, by promoting proliferation, angiogenesis, and biomineralization through enhanced gene activation; this could translate into effective pulpal repair and more predictable formation of reparative dentin [43].

## 5. Conclusion

Within the limitations of this study, the Single Bond Universal appears to remain inappropriate for human pulp capping; further long-term studies are needed to determine its biocompatibility.

## Figures and Tables

**Table 1:** Composition of the tested materials according to the manufacturers.

Material	Composition	Mode/steps of application
Calcipast (Cerkamed, Poland)	Calcium hydroxide, barium sulfate, hydroxyapatite, propylene glycol	Apply the material directly to the pulp
Single Bond Universal (3M ESPE, Germany)	HEMA, MDP, dimethacrylate resin, photoinitiator system, methacrylate modified polyalkenoic acid copolymer, filler, water, ethanol, silane	Apply a thin layer of the adhesive to the enamel, dentin, and pulp with the applicator and allow it to act for 20 seconds; dry the adhesive layer for at least 5 seconds; polymerize with blue light for 10 seconds

HEMA: 2-hydroxyethyl methacrylate; MDP: methacryloyloxydecyl dihydrogen phosphate.

**Table 2:** Scores used during the histological analyses of the calcific barriers and dental pulp.

Scores	Calcific barrier continuity
1	Complete dentin bridge formation
2	Partial/incomplete dentin bridge formation extending to more than one-half of the exposure site but not completely closing the exposure site
3	Initial dentin bridge formation extending to not more than one-half of the exposure site
4	No dentin bridge formation

Scores	Calcific barrier morphology

1	Dentin or dentin associated with irregular hard tissue
2	Only irregular hard tissue deposition
3	Only a thin layer of hard tissue deposition
4	No hard tissue deposition

Scores	Calcific barrier thickness

1	>0.25 mm
2	0.1–0.25 mm
3	<0.1 mm
4	Absent bridge

Scores	Inflammation type

1	No inflammation
2	Chronic inflammation
3	Acute and chronic inflammation
4	Acute inflammation

Scores	Inflammation intensity

1	Absent or very few inflammatory cells
2	Mild (an average of <10 inflammatory cells)
3	Moderate (an average of 10–25 inflammatory cells)
4	Severe (an average of >25 inflammatory cells)

Scores	Inflammation extensity

1	Absent
2	Mild (inflammatory cells next to dentin bridge or area of pulp exposure only)
3	Moderate (inflammatory cells observed in one-third or more of the coronal pulp or in the midpulp)
4	Severe (all of the coronal pulp is infiltrated or necrotic)

Scores	Odontoblastic cell layer

1	Palisade pattern of cells
2	Presence of odontoblast cells and odontoblast-like cells
3	Presence of odontoblast-like cells only
4	Absent

Scores	Presence of bacteria

1	Absence
2	Presence of stained bacterial profiles along the coronal or apical walls
3	Presence of stained bacterial profiles within the cut dentinal tubules or axial wall
4	Presence of stained bacterial profiles within the dental pulp
